# Sustainability and Equity in Urban Development (S&EUD): A Content Analysis of “Bright Spots” from the Accelerating City Equity (ACE) Project

**DOI:** 10.3390/su15097318

**Published:** 2023-04-28

**Authors:** Nishita Dsouza, Anitha Devadason, Araliya M. Senerat, Patrin Watanatada, David Rojas-Rueda, Giselle Sebag

**Affiliations:** 1 International Society for Urban Health (ISUH), New York, NY 10003, USA; 2 Social Intervention Group, Columbia University School of Social Work, New York, NY 10027, USA; 3 Urban Health Collaborative, Dornsife School of Public Health, Drexel University, Philadelphia, PA 19104, USA; 4 Colorado School of Public Health, Colorado State University, Fort Collins, CO 80532, USA

**Keywords:** equity, sustainability, urban development, content analysis, implementation

## Abstract

Sustainable and equitable urban development (S&EUD) is vital to promote healthy lives and well-being for all ages. Recognizing equity as core to urban development is essential to ensure that cities are inclusive, safe, resilient, and sustainable. The aim of this study was to identify and assess the elements of equity and sustainability in exemplary bright spots using the ACE Framework and the United Nations’ 5 Ps of Sustainable Development. A content analysis process was performed to identify initial case studies, obtain bright spot information, and select final case studies. The exemplary bright spots selected were assessed for drivers of equity and the five pillars of sustainability. Results showed that equity and sustainability have become key considerations in urban development work. Numerous effective strategies and outcomes identified in the exemplary bright spots could be replicated in other contexts.

## 1. Introduction

Cities and urban development are complex, dynamic, and systemic phenomena, the positive and negative consequences of which are addressed in the United Nations 2030 Agenda for Sustainable Development [1]. Scholars assert that urban development cannot be truly sustainable without addressing inequities in all of their forms and that initiatives that do not center equity will perpetuate economic and social inequalities, and uneven power relationships and politics [2]. Equity is naturally implied in global definitions of sustainability; for example, the 1987 Brundtland definition—”meeting the needs of the present without compromising the ability of future generations to meet their own needs”—hinges on the concept of intergenerational equity [3]. However, equity is not uniformly integrated into sustainable urban development initiatives due to a greater focus on outcomes versus if the outcomes are distributed fairly, fairness of processes or the equity of underlying structures and systems affecting outcomes [2,4–6]. There is much overlap between the various dimensions of equity and sustainability, and due to the large focus on outcomes, there is a significant gap in implementation science research on how to integrate equity in sustainable urban development.

There is a limited understanding of the processes and elements that lead to sustainable and equitable urban development (S&EUD), and as a result, equity has not been main-streamed into sustainable development policy and practice. Additionally, there is a dearth of evidence on how to make sustainable development an engine for improved equity and social justice [7–12]. However, cities and local communities are applicable settings for effective responses to these challenges, as populations experience and interpret inequity as part of their livelihoods and seek creative and pertinent ways of responding to these circumstances that negatively impact their health and well-being.

There are strong connections between environmental health and issues of justice and equity, necessitating a holistic conceptualization of healthy urban design and planning [13]. Equity is core to sustainable development, and sustainable development cannot be fully achieved without equity. The United Nations’ Sustainable Development Goals (SDGs) reference the importance of equity on multiple occasions. To ensure that cities are inclusive, safe, resilient, and sustainable (SDG 11) and ensure healthy lives and well-being for all ages (SDG 3), recognizing equity as core to sustainable development is critical. We define equity as the fair and just distribution of resources and opportunities to all population groups, particularly marginalized populations, within and between communities [14]. Additionally, we define health equity within sustainable urban development to mean all population groups and communities have an equal/fair/just opportunity to support physical and social environments, allowing them to achieve optimal health today, while also sustaining opportunities for future generations. Lastly, we define equity in sustainable development (ESD) as all community members, especially those that have been historically under-resourced, having agency over and equitable access to environments and opportunities that support and enhance health and wellbeing today and for future generations. To reduce inequity and health inequalities in cities, a recognition of equity in both physical and social environments should be incorporated to ensure that outcomes promote health for everybody in society, not only those with the most agency and power [13]. While there is much theoretical knowledge about S&EUD, it is still an understudied concept and urban health is a growing field [2–6]. There is much potential for implementation science theories, frameworks, and methods to advance scientific knowledge on urban health and support implementation processes of S&EUD on the ground.

**Figure 1.**
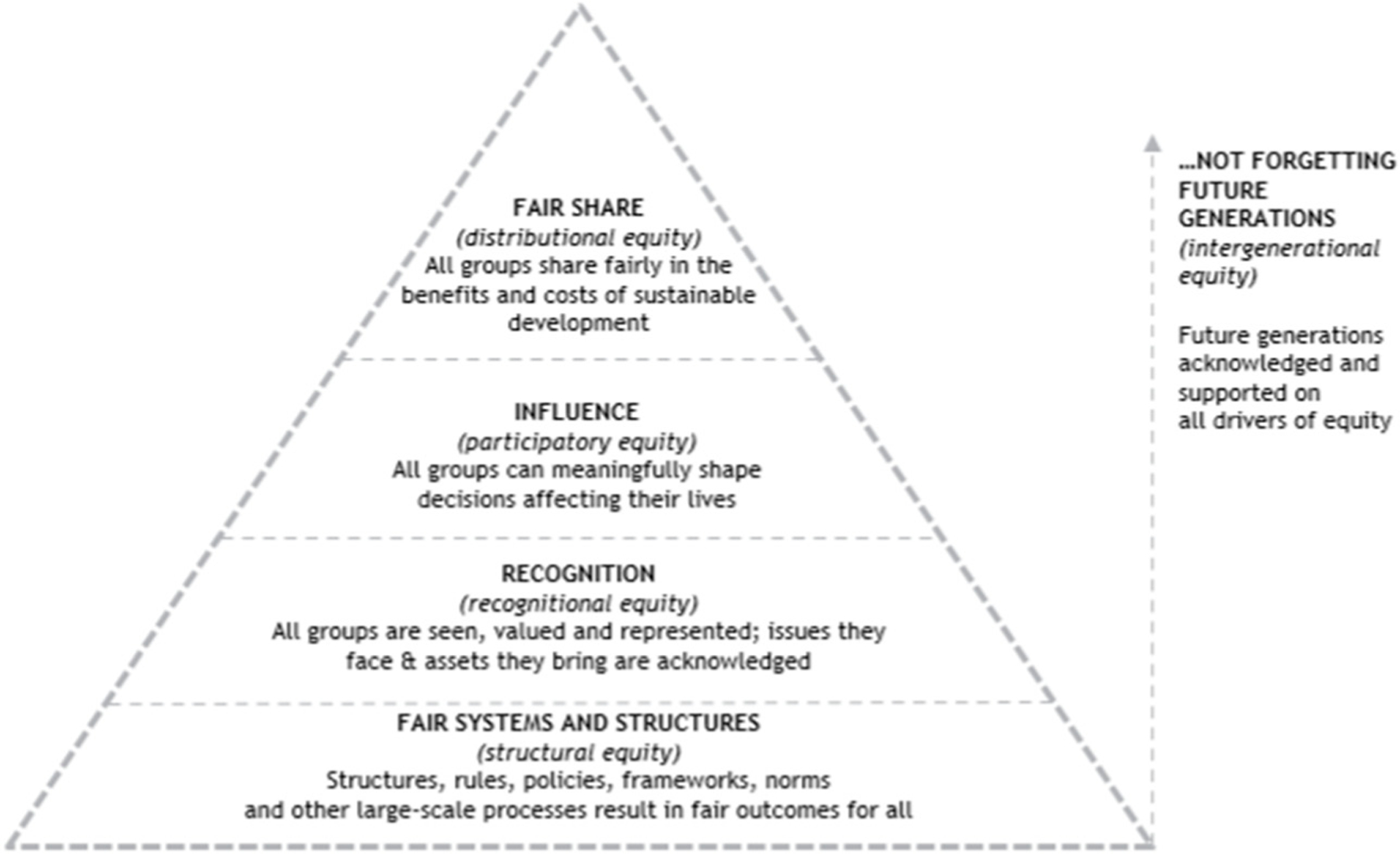
Accelerating City Equity (ACE) Framework: Five Dimensions of Equity.

The Accelerating City Equity (ACE) project is a global knowledge exchange project, with the aim of establishing a body of knowledge between the intersection of equity and sustainability to grow a community of development practitioners that will drive the implementation of S&EUD projects globally. The ACE Framework (Figure 1) was created by the International Society for Urban Health (ISUH) to improve urban inequity within sustainable development around the world [14]. Our concept of equity was operationalized as a framework encompassing five dimensions: Recognitional Equity (recognizing and understanding that populations have different histories and needs); Procedural Equity (all population groups must participate in decision-making processes that affect them); and Distributional Equity (goods, services, and opportunities must be distributed equitably to all stakeholders).

In addition to equity, sustainability in urban development is also a core focus as climate impacts are experienced inequitably globally by low-resource populations [15]. The 5Ps from the United Nation’s (UN) 2030 Agenda is a global framework with five “pillars” of sustainable development [1,16], including:

- People: ensuring all people are receiving the proper resources and need to maintain a healthy livelihood
- Planet: slowing the progression of climate change
- Peace: having peace between and within countries
- Prosperity: economic growth within societies and countries
- Partnerships: partnerships between countries to encourage development

Existing articles on equity and sustainability range in various aims, including emphasizing the importance of sustainability in future projects [11], identifying challenges and evaluating current ways equity and sustainability are achieved [8,17–19], and recommending strategies for incorporating equity and sustainability [7,20]. This area may also range in a variety of fields across the health and environment intersection [9,20,21]. One project, EU Horizon 2020 INHERIT project (2016–2019), had a similar approach to identifying policies and practices that contribute to health, equity, and environmental sustainability [20,22]. While this project identified numerous lessons learned for good practices to achieve health and environmental sustainability, the focus was on only one aspect of sustainability within Europe. To our knowledge, there have been no studies identifying and evaluating equity and the five Ps of sustainability on existing successful projects. This gap needs to be filled to better translate existing lessons learned from global bright spots to other local contexts.

The aim of this study was to identify and assess the elements of equity and sustainability within exemplary bright spots, and urban health initiatives across the globe aimed at improving disparities (see Table 1). This research will inform our understanding of the processes used in S&EUD, with the eventual goal of documenting lessons learned and replicating these processes and eventual outcomes in other urban contexts. We hypothesize that numerous existing bright spots will touch on equity and sustainability and that lessons from each can be identified and translated into other contexts.

## 2. Materials and Methods

**Figure 2.**
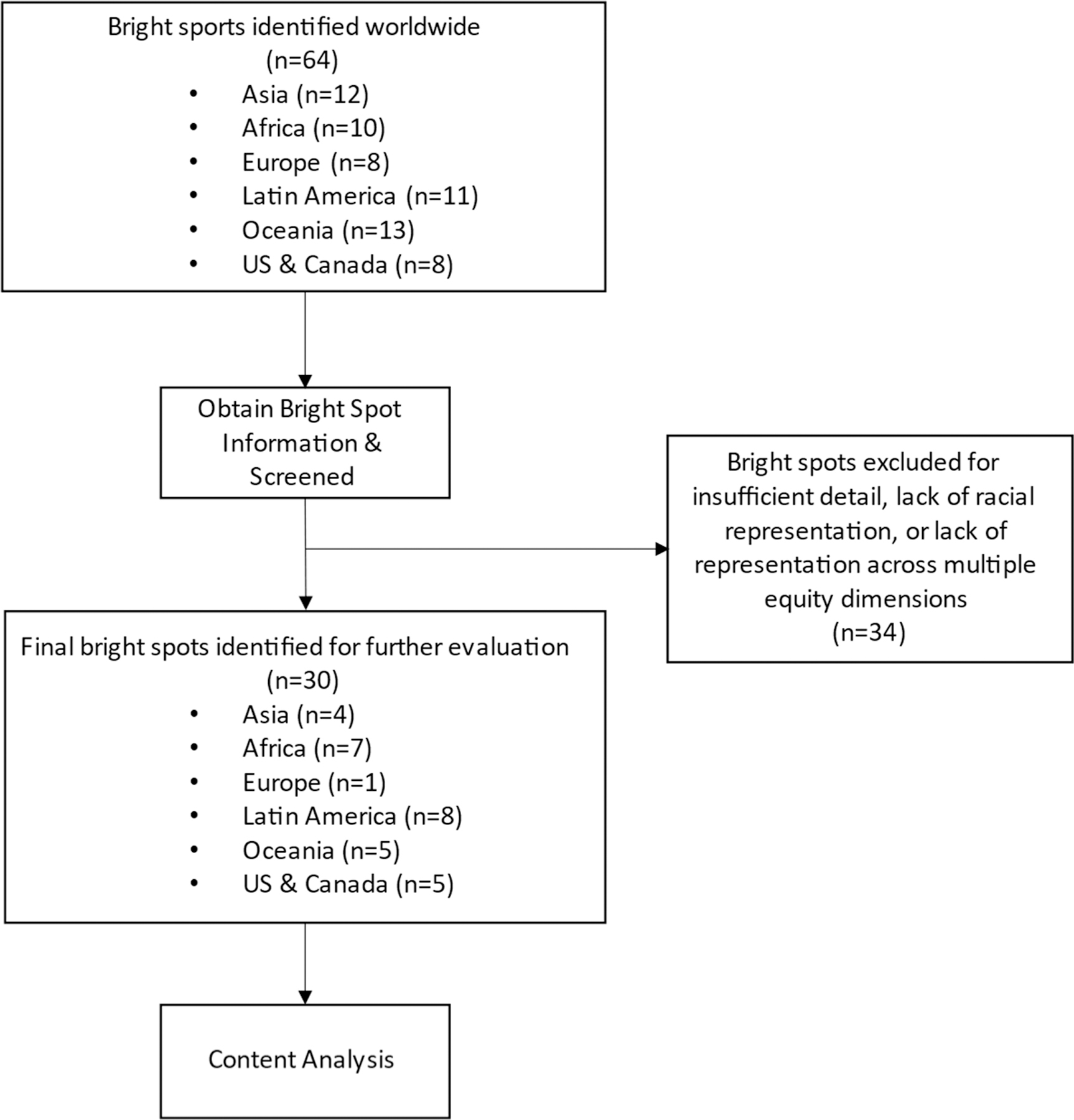
Flow chart for identifying and selecting bright spots.

The content analysis process was divided into four steps: (i) identifying initial case studies, (ii) obtaining bright spot information; (iii) selecting final case studies, and (iv) final analysis. Figure 2 provides a flow chart of the methodology around identifying and selecting these case studies.

### 2.1. Identifying Initial Case Studies

Case studies, projects, and practical examples (referred to as bright spots) that drive equity in sustainable development were identified, within the six ACE regional hubs that were created (Africa, Asia, Oceania, Europe, Latin America, and North America). These ‘bright spot’ case studies were presented by members of local community-based organizations, government, private sector companies, researchers, and/or civil society groups.

Bright spots were defined as a case study that aims to improve environmental, system-level, community-level, and/or individual-level disparities affecting health and well-being outcomes of historically underserved groups, either directly or within universal approaches and demonstrates sustained impact over time with transferable learnings. Bright spots were identified using a variety of methods, including the identified existing tools, newsletters to which members and authors subscribed, word-of-mouth, and projects with which regional hub members were familiar.

### 2.2. Obtaining Bright Spot Information

Once bright spots were identified, a Deeper Dive Exploration Guide was sent to regional hub members to collect information on the following areas: sustained outcomes, context, benefiting groups, key decision-makers and actors, goals, location, timeframe, health, and well-being issues addressed, key strategies or actions, budget, and results. Once Exploration Guides were collected, bright spots were selected by the ISUH team for further analysis based on those that had any missing information not yet identified.

After this primary identification, one-on-one interviews were conducted by the ACE project team with regional hub members to learn more about the bright spots. These interviews were semi-structured using the Deeper Dive Exploration Guide as an interview guide to fill in any gaps that were missing from the survey document. Interviews were conducted on a virtual meeting platform (e.g., Zoom, Microsoft Teams), and lasted about 30 min to one hour each. Members provided as much sufficient information as they could regarding the projects.

### 2.3. Selecting Final Case Studies

Exemplary bright spots were chosen for additional analysis based on the following criteria:

- Representation of multiple dimensions of equity
- Geographical diversity
- A scale incorporating both a bottom-up and top-down approach
- Sufficient information provided
- Recurring themes
- Racial representation

Two ISUH team members reviewed and screened the content of the bright spots provided by the regional hub members to select the exemplary ones that were the most equitable and sustainable, while a third team member voted on any discrepancies between the two team members. Many of the bright spots did not have sufficient detail compared to others and therefore were excluded from the content analysis stage.

### 2.4. Content Analysis

After the selection of the final exemplary bright spots, a content analysis was conducted by reviewing the information provided by the Deeper Dive Exploration Guides, interviews with regional hub leaders and ACE members, and supporting project materials submitted to the ACE team. Bright spots were categorized by major theme, and descriptive information about their location and the timeframe was tabulated. The ACE Framework Five Dimensions of Equity and the UN’s 5 Ps of Sustainability were used as guiding conceptual frameworks, to capture the interdependencies between the various types and domains of these two concepts. Two ISUH team members reviewed each of the exemplary bright spots to identify the equity and sustainability strategies using these two frameworks. Transferable strategies were identified and summarized.

## 3. Results

A total of 64 bright spots were identified globally. From the defined criteria for selecting and highlighting exemplary bright spots to showcase, a total of 30 bright spots were identified for content analyses (Table 1). Exemplary bright spots were most often identified or categorized along the basis of infrastructural/sectoral services, such as housing (*n* = 4), governance (*n* = 4), water and sanitation/hygiene (*n* = 4), food systems and/or agriculture (*n* = 3), waste management and recycling (*n* = 2), access to income and/or work (*n* = 1), and healthcare (*n* = 1). Bright spots were also categorized by built and natural environment foci, such as placemaking (*n* = 2) and climate change (*n* = 2). Lastly, exemplary bright spots were categorized by special populations, such as early childhood (*n* = 2), gender equity (*n* = 2), and racial equity (*n* = 3). Even though they were categorized in these buckets primarily, there is much overlap in categories (e.g., the exemplary bright spot of Mahila Housing Trust primarily focuses on housing but has a strong gender equity component).

### 3.1. Location and Timeframe of Exemplary Bright Spots

The exemplary bright spots ranged in different locations: Oceania (*n* = 4), Asia (*n* = 4), Europe (*n* = 1), Latin America (*n* = 8), USA/Canada (*n* = 5), and Africa (*n* = 8) (see Table 1). Exemplary bright spots were identified in both LMICs and HICs, representing much global diversity in contexts. Many of the exemplary bright spots, 26 out of 30, are still ongoing, whether through programmatic efforts or policy ramifications.

### 3.2. Equity Dimensions Identified in Exemplary Bright Spots

Table S1 describes the transferable tools and strategies identified in the exemplary bright spots across the five dimensions of equity from the ACE Framework. In summary, 14 bright spots touched on all five dimensions of equity, with the remaining touching on at least 2 dimensions of equity. These are further explored in the sections below.

#### 3.2.1. Distributional Equity

**Figure 3.**
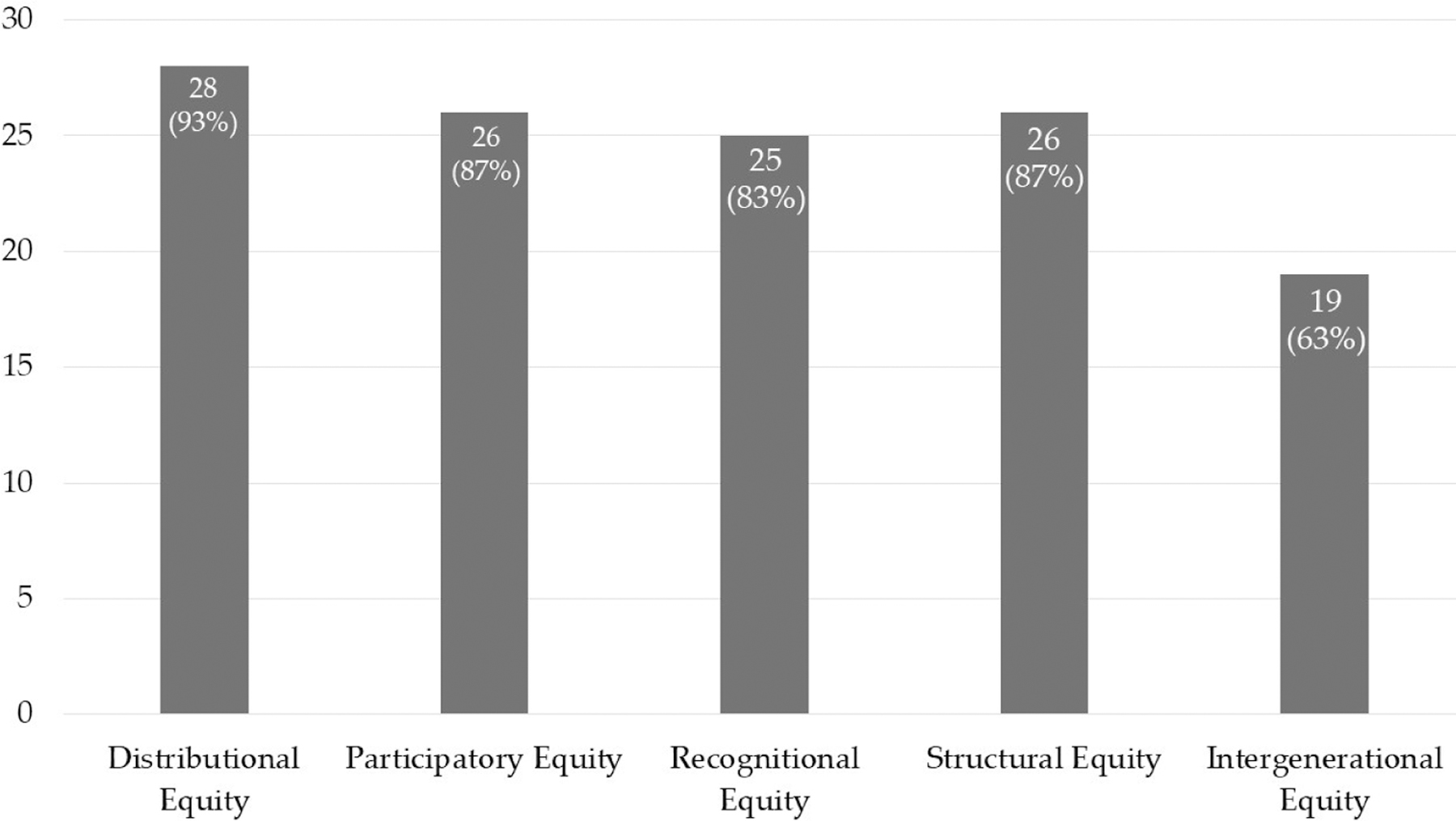
Equity Dimensions Identified in Exemplary “Bright Spots”.

Distributional equity is defined as the increase or fairer distribution of urban underserved groups’ access to the benefits of, and/or reducing their share of the costs or burdens of, urban infrastructure, resources, policies, programs, services, amenities, nature, etc. Most exemplary bright spots, 28 of the 30, discussed distributional equity (see Figure 3).

Exemplary bright spots ranged in how they achieved distributional equity, with many discussing the direct impacts on community residents. For example, multiple exemplary bright spots discussed changes in more tangible goods and services, such as the provision of infrastructural services or systems (e.g., housing, water, and sanitation services). Other exemplary bright spots focused on human capital, such as changes in opportunity (e.g., employment options or social entrepreneurship) or the building of knowledge or skills (e.g., technical literacy, creation of social cooperatives).

Some exemplary bright spots discussed distributional equity in more indirect terms, such as framing policy changes as achieving distributional equity through downstream impacts. For example, the Healthy Liveable City policy and spatial indicators research program discussed distributional equity in the impact it had on decision-makers increasing their knowledge base of cost-effective interventions for this population. Another similar example was the Advancing Racial Equity on Planning and Policy Toolkit, which discussed how the toolkit was integrated into project development processes and implied how long-term infrastructure investments would be more fairly distributed.

Lastly, exemplary bright spots discussed achieving distributional equity as a theoretical end result. For example, the Observatory of Urban Health of Belo Horizonte (OSUBH) discussed how the program was implemented to promote physical activity in vulnerable communities, connecting data provisions and the actual impact of increasing physical activity among the target population.

#### 3.2.2. Participatory Equity

Participatory equity is defined as increasing the influence or power of the group over decisions that affect them. Most exemplary bright spots, 26 of the 30, discussed participatory equity (see Figure 3).

Exemplary bright spots ranged in the distribution of the groups that were involved in initiating and leading decisions over the community. Over half (14/26) of exemplary bright spots exhibited stakeholders other than the local community acting as a bridge to initiate and mobilize action within the community. These included researchers or academia conducting initial training, local government such as the Parks and Recreation Department in the Measure A Initiative in Los Angeles, CA, USA hiring local community organizations to host local community meetings to ask what the community desires or local grassroots organizations acting as a bridge between residents and the private sector as with the Mahila Housing Trust in India.

Robust participatory equity was identified in about 40% (11/26) of exemplary bright spots where residents of communities organized themselves to carry out various actions ranging from community mapping, organizing themselves into committees, and pushing for policy change. Key to participatory equity was the recognition that initiatives were inclusive in involving those who have a mutual understanding, as a lack of group cohesion can impede efforts. This was exemplified in the Herbal and Nutrition Garden in Warren Park, where sustainability was not maintained as a lack of group cohesion was a huge barrier to the continuation of the initiative beyond the donor-funded period.

#### 3.2.3. Recognitional Equity

Recognitional equity is defined as increasing the status, legitimacy, recognition of, representation of, or respect for the group’s existence, assets, needs, rights, or vulnerabilities. The most exemplary bright spots, 25 of the 30, discussed recognitional equity (see Figure 3). This was performed through a variety of methods, including conducting research to identify problems to tackle, having community-driven research conducted, or identifying existing problems already known within the region.

The majority of the recognitional strategies were obtained through research, including investigator- or community-driven research. The Mahila Housing Trust in Asia, for example, involved community women to act as mobilizers and identify problems by interviewing family, friends, and neighbors. The Local Play Every Day project in Oceania also had a similar strategy in having the community take the lead in addressing health concerns for their children by securing child-led free play.

Other bright spots used a more academic approach. For example, Re-ciclo, a Latin America-based project, conducted focus groups and technical visits to understand the demands of waste collectors in Fortaleza, Brazil prior to providing electric cargo tricycles to assist waste collectors in collecting recyclable materials around the community.

Lastly, other bright spots used already existing data and knowledge to recognize problems within the community. The Long Beach Fresh Crop Swap of the USA and Canada region used existing data to identify the three neighborhoods in Long Beach that reported the highest levels of obesity, diabetes, and asthma. This knowledge was then used to target residents in this neighborhood with a fresh crop swap to encourage healthy eating. The Urban95 Crezco con mi Barrio (Grow with my Neighborhood) used existing data to create heatmaps to identify neighborhoods where young children face the greatest challenge in Bogotá. This allowed for child-friendly public spaces to be targeted to areas that are needed the most.

#### 3.2.4. Structural Equity

Structural equity is defined as changing institutional rules, policies, practices, social norms, mental frameworks, or other structural factors or large-scale processes that contribute to and perpetuate pre-existing inequalities in power, wealth, underlying health, exposure to risks, etc. Many exemplary bright spots, 26 of the 30, discussed structural equity (see Figure 3).

Many discussed the policy or infrastructural changes resulting from their project or initiative, such as new taxation systems, lawn ownership, housing standards, amendments to city plans, regulatory or financial oversight, etc. It is important to note that the policy focus was not always the main objective of the bright spot, but that the overall project resulted in significant policy changes that achieved structural equity.

Additional exemplary bright spots discussed changes to social norms that resulted in structural equity, such as the formation of cooperatives, social agreements focused on savings, etc. These changes weren’t only found at the individual level, but also at the institutional level, with two exemplary bright spots discussing changes made to funding organizations. Additional bright spots discussed how social norms changed at the policy level, with governmental actors using data in new ways, or structurally reorganizing civic administration to involve women in governance.

#### 3.2.5. Intergenerational Equity

Intergenerational equity is defined as supporting structural, recognitional, participatory, and/or distributional equity for future generations. Less than half of the exemplary bright spots, 19 of the 30, discussed intergenerational equity (see Figure 3). While none of these bright spots explicitly discussed how they focused on its recognitional element, all of them touched on how they centered or amplified the voices or priorities of children or future generations.

The structural component of Intergenerational equity was discussed by 3 exemplary bright spots. Examples included changing social norms to emphasize safety for children and future generations, expanding training or job opportunities for children or future generations, and focusing on children in training or life skill development.

The distributional component of intergenerational equity was touched on by 3 exemplary bright spots, all of which discussed it in the context of new amenities, such as road infrastructure or public spaces, that would benefit kids and future generations.

Lastly, the participatory component of intergenerational equity was discussed by 2 exemplary bright spots, which involved kids in decision-making or increasing the participation and engagement of children in the program or initiative.

### 3.3. Pillars of Sustainability Identified in Exemplary Bright Spots

Table S2 describes the dimensions of sustainability identified in the exemplary bright spots across the “5Ps” from the United Nations (UN) 2030 Agenda: people, planet, profit, peace, and partnerships. In summary, 12 bright spots touched on all five pillars of sustainability, with the remaining touching on at least 3 pillars of sustainability.

#### 3.3.1. People Pillar (Social Sustainability)

The people pillar of sustainability, also known as social sustainability, refers to the outcomes of ensuring inclusion and adequate human development opportunities for populations. The SDGs refer to this pillar as the world’s determination “to end poverty and hunger, in all their forms and dimensions, and to ensure that all human beings can fulfill their potential in dignity and equality and in a healthy environment” [16]. All exemplary bright spots touched on social sustainability in some way (see Figure 4).

One of the most common mechanisms through which exemplary bright spots discussed social sustainability was through improvements to individual and community health. Of the 30, 13 exemplary bright spots mentioned improving health through a myriad of ways, such as less diarrheal disease through improved hygiene practices, increased mental health through reduced feelings of isolation and increased social inclusion, or increased physical activity through the creation of active living opportunities or improved built environment infrastructure.

Another common theme of exemplary bright spots centering social sustainability was through the strengthening of social or network ties and the building of social capital. Of the 30, 12 exemplary bright spots discussed how they resulted in greater community linkages and opportunities for social connection, stronger systems of caregiving, fostered the inclusion of traditionally marginalized populations in decision-making and included the voices of traditionally marginalized populations.

Several bright spots, 4 out of 30, discussed increased security as a result of their efforts, whether through housing ownership and land tenure, increased financial security, or improved perceptions of community safety. A few bright spots also touched on self-empowerment and community empowerment through entrepreneurial activities.

#### 3.3.2. Planet Pillar (Environmental Sustainability)

The planet pillar of sustainability, also known as environmental sustainability, refers to engaging in climate action. The SDGs that are relevant to this pillar share a goal to protect the planet “so it can support the needs of the present and future generations” [16]. Of the 30 exemplary bright spots, 21 of them discussed environmental sustainability (see Figure 4).

The commonly mentioned theme was waste management, with 8 exemplary bright spots discussing this. The projects focused on water and sanitation/hygiene issues of human waste management, trash dumping, or commercial or vehicular air pollution. Water management was also discussed, with 5 exemplary bright spots. Projects focused on reducing flooding, stormwater management, cleaning waterways or blue space conservation, and conserving ecology for wildlife.

Another frequently mentioned approach for achieving environmental sustainability was greenspace creation or urban greening, with 5 exemplary bright spots focused on this topic conducting landscaping interventions, urban agricultural programs, improved livestock cultivation, and tree planting. Less mentioned approaches to environmental sustainability included focusing on recycling or reusing materials, sustainable energy, and urban heat management.

#### 3.3.3. Profit Pillar (Economic Sustainability)

The profit pillar of sustainability, also known as economic sustainability, is about supporting growth, jobs, and poverty reduction. The SDGs specific to this pillar aim to “ensure that all human beings can enjoy prosperous and fulfilling lives and that economic, social, and technological progress occurs in harmony with nature” [16]. Of the 30 exemplary bright spots, 12 discussed economic sustainability (see Figure 4).

The most often discussed method through which exemplary bright spots (7 out of 30) addressed economic sustainability was through directly fostering business or entrepreneurial skills with community residents. Projects or initiatives discussed setting up of home-based businesses and independently owned shops, creation, or connection to skill-building apprenticeships (in both private and public sectors), teaching grant writing skills and government contract procurement, and other business models to foster entrepreneurship among the target population.

Another common theme among exemplary bright spots, discussed by 4 out of the 30, was communal or collective savings efforts, such as the creation of cooperatives, community tourism business models, or urban agricultural initiatives. Additional bright spots discussed structural efforts for intergenerational wealth building or the creation of financial sustainability, such as the provision of low-interest housing loans or the creation of community-owned waste management systems.

#### 3.3.4. Peace Pillar (Peace Sustainability)

The peace pillar of sustainability focuses on strengthening institutions and governance and tackling corruption. The SDGs specific to this pillar affirm that “there can be no sustainable development without peace and no peace without sustainable development” [16]. Of the 30 exemplary bright spots, 18 discussed peace sustainability (see Figure 4).

The most frequently mentioned was gender equity (7 of 30 exemplary bright spots), followed by a focus on unhoused or housing insecure populations (5 of 30), racial and ethnic minority populations (5 of 30), children (4 of 30), individuals living with a disability (2 of 30), indigenous populations (1 of 30), and immigrants and refugees (1 of 30). Most exemplary bright spots discussed the empowerment and equity of these populations broadly, and strategies such as economic empowerment, involvement in political decision-making, or inclusion in governance.

#### 3.3.5. Partnerships Pillar (Collaborative Sustainability)

The partnerships pillar of sustainability, also referred to as collaborative sustainability, emphasizes how global SDGs are financed and upheld across national and international actors. Relevant SDGs call for “a spirit of strengthened global solidarity” [16]. This was the second most mentioned pillar of sustainability with 29 out of the 30 exemplary bright spots discussing collaborative sustainability (see Figure 4).

Collaboration across governmental entities at multiple levels (e.g., local and state levels, or local and federal levels) were referenced most often, with 12 of the 30 exemplary bright spots discussing how programs were initiated or sustained. This was closely followed by the collaboration of public-private partnerships or engagement of public-private partnerships with community residents (discussed in 11 of 30 exemplary bright spots). After that, multisector partnerships, or collaboration across different public sectors or departments (e.g., housing, transportation, health) were discussed in 9 of 30 exemplary bright spots. Partnerships between government entities and/or private organizations with international aid agencies or funders were discussed in 8 of 30 exemplary bright spots, most often in the context of program piloting or sustenance. Local universities were discussed as a relevant collaborative partner in 3 of the 3 bright spots, and only one exemplary bright spot discussed the collaboration of community residents across class lines (e.g., lower-class community residents supported by middle-class professionals in relevant sectors such as architecture or law).

## 4. Discussion

The aim of this analysis was to investigate exemplary bright spots identified in the ACE project for elements of equity and sustainability and to examine how these elements overlapped or were leveraged to drive forward S&EUD. Our hypothesis was correct in that many of the existing bright spots had strategies aiming to achieve equity and sustainability within the community. In addition, a variety of lessons learned were pulled from each bright spot that can be used by future projects to achieve S&EUD. Identified bright spots were truly local-to-global in nature, with identifiable and transferable lessons learned across urban contexts, despite the diverse locations in which these projects and policies were implemented. Scholars advocating for the decolonization of global health have identified local-to-global approaches to bidirectional exchanges of knowledge as a method to deviate from the assumed superiority of the Global North over the Global South [23]. The ongoing nature of many projects signifies great commitment among invested stakeholders in sustaining these initiatives and ensuring equity and sustainability targets are met.

Overall, the exemplary bright spots identified ranged in various themes of infrastructural/sectoral services, built and natural environment foci, and special populations. Of the 30 exemplary bright spots examined, 14 used all five dimensions of equity, and 12 used all five pillars of sustainability. The most common dimension of equity mentioned was distributional equity and the least common was intergenerational equity. The most common pillar of sustainability was the people pillar or social sustainability and the least common is the profit pillar or economic sustainability. These S&EUD elements identified within these bright spots are important lessons learned that future bright spots can use to apply actionable strategies to promote equity and sustainability within the community, and could particularly be beneficial to promote the health and well-being of those in marginalized communities.

One essential finding from this content analysis is the dual nature of both equity and sustainability as outcomes, but also processes. These have long been understood as separate, moving targets. The content analysis of exemplary bright spots identified in the ACE project depicts the close intertwining of two overlapping yet conceptually distinct concepts in urban development: equity and sustainability. This paper shows how the exemplary bright spots touch on both the dimensions of equity and the pillars of sustainability in separate instances, yet these strategies of bright spots have similar goals in promoting equity within sustainability. Previous studies have emphasized the importance of sustainability and equity in a variety of projects focused on specific topics, such as the environment [17,24], food systems [21], etc. To the authors’ knowledge, no paper has been published to assess current bright spots that focus on a range of topics related to both topics at the urban level, all of which share common causal pathways, intervention points, and levels that can be examined from systems science approach [13,25].

Of the five dimensions of equity, intergenerational equity was discussed least often, largely attributable to its nature as a lag measure, and the lengthy amount of time needed to assess impacts across generations [26]. However, most projects will likely have intergenerational impacts if achievements in other dimensions of equity are sustained.

The people pillar, or social sustainability, was the most articulated element, most often in the context of how projects or initiatives improved the health of individuals and communities. This is largely due to the nature of this project and the audience it targeted (i.e., urban health professionals). Conversely, the profit pillar or economic sustainability was the least articulated element. This is largely attributable to the diversity of exemplary bright spots, and their foci on topics other than opportunity creation or wealth generation, or their inability to measure the economic impact of the bright spot, either over time or as a spillover effect.

A common theme woven through exemplary bright spots was the importance of partnerships or collaborative sustainability. S&EUD concepts typically span multiple sectors, transcending political divides and ideological differences to translate knowledge into action [27]. One of the mechanisms through which S&EUD implementation is driven forward is the process of storytelling and framing of urban investments into simplified, compelling narratives depending on the audience they are persuading [28]. Future ACE project goals include translating several exemplary bright spots into storytelling resources, centering and amplifying the voices of community residents to depict project impacts beyond quantitative measurement.

One of the ACE project goals was this concept of “accelerating” the process of urban equity, by better understanding the mechanisms by which equity and/or sustainability were achieved, to essentially speed up the process. Documentation of these best practices and the processes in which successful outcomes resulted in exemplary bright spots is an essential step to translating this information to other contexts and replicating these best practices. Future planning of bright spots should ensure that projects should aim to touch on all aspects of equity drivers and sustainability pillars to be successful. Future efforts of the ACE project involve updating the ACE Equity Framework in response to regional hub leaders’ and project members’ feedback to lessen its focus on academic concepts and to increase its utility as an assessment tool.

Future ACE project initiatives are designed to fill much-needed gaps in the translation of S&EUD concepts into practice. While S&EUD conceptual frameworks are being developed, further implementation science research is needed to better understand how these concepts are operationalized at individual, organizational, community, and policy levels. One of the next steps of the ACE project involves the creation of an online knowledge exchange platform, which can contribute to much-needed knowledge generation around the implementation science of S&EUD interventions [13].

Transferrable lessons learned from S&EUD case studies are applicable to a wide range of audiences, including but not limited to community leaders and grassroots organizations, city policymakers and departmental leaders, built environment practitioners, public health professionals, and academic researchers. We recommend future policies to encompass both equity drivers and sustainability pillars to ensure its’ success and reach all individuals.

### Strengths and Limitations

The paper herein has many strengths. Firstly, this assessment of bright spots from the ACE Project includes a vast array of projects, ranging in location, participants targeted, and areas of expertise. As an international organization, ISUH was able to use its international network to gather members to share their suggested bright spots. This allowed ACE to include a diverse range of bright spots to learn from. Secondly, the bright spots chosen were focused on its success, and therefore we were able to pull successful strategies from these projects to align along both the drivers of equity and sustainability pillars. Thirdly, using both the ACE Framework and the UN’s 5 pillars allowed us to compare equity and sustainability, respectively, providing key insights into how these and future bright spots can be assessed for equity and sustainability using these existing tools and frameworks. In addition, the next steps of the ACE Project include updating the framework based on feedback from the members and creating a knowledge exchange platform to share existing bright spots across networks.

The results reported should be considered considering some limitations. Limitations were noted in the collation of bright spots. While 30 exemplary bright spots were selected out of a total of 64 for this analysis, the regional spread lacked in one region. Exemplary bright spots from Europe were lacking and had only one exemplary bright spot, compared to those from other regions across Asia, Africa, Oceania, North America, and Latin America, limiting regional representation of the data in Europe alone. However, given that previous work had already been conducted in Europe with the EU Horizon 2020 INHERIT project (2016–2019) mentioned above, we do not believe that geographic representation is unbalanced as bright spots in Europe were already identified through another project and that there is more to learn from bright spots in the rest of the world. The goal of the ACE project is the bidirectional exchange of knowledge, and European partners that are currently engaged with this project have learned much from bright spots in other parts of the world, especially the Global South, which historically has had less focus on urban health research. Additionally, information gathered for the bright spots was reliant on the provision of information from various participants, some of whom were not explicitly involved in the bright spots themselves or were tangentially involved. These included participants who were no longer involved in an ongoing bright spot, or who had never been involved in the bright spot directly. As a result, clarifying information was not always readily available, thus limiting access to data. To reduce this limitation, survey instruments in the form of a Deep Dive template form were provided to all participants to collect data; however, the depth and completion of the information varied across bright spots. Lack of available or reliable data resulted in the selection of bright spots with greater depth of information for analysis, resulting in subjective identification of bright spots. This lack of sufficient information was the biggest barrier to why only one bright spot was chosen as exemplary in Europe. This may have limited the regional distribution of selected bright spots and the selection criteria of bright spots including the number of exemplary bright spots exhibiting the most dimensions of equity.

## 5. Conclusions

Our analysis has shown that equity and sustainability have become key considerations in urban development work. As part of the ACE project, we examined a range of case studies that integrate equity and sustainability elements. Thirty exemplary bright spots were identified, ranging in various themes of infrastructural/sectoral services, built and natural environment foci, and special populations. Fourteen of the bright spots touched on all five dimensions of equity and twelve of the exemplary bright spots touched on all five pillars of sustainability, with the most common being distributional equity and the people pillar, or social sustainability and the least common being intergenerational equity and profit pillar, or economic sustainability. Implementation science research approaches are increasingly leveraged for healthy urbanism and the advancement of S&EUD [29]. Through this analysis, we have identified numerous effective strategies and outcomes that could be replicated in other contexts. Future studies should examine the implementation of these transferable lessons learned and identify challenges that one may face when S&EUD strategies.

## Supplementary Material

Table S1 and Table S2

## Figures and Tables

**>Figure 4.**
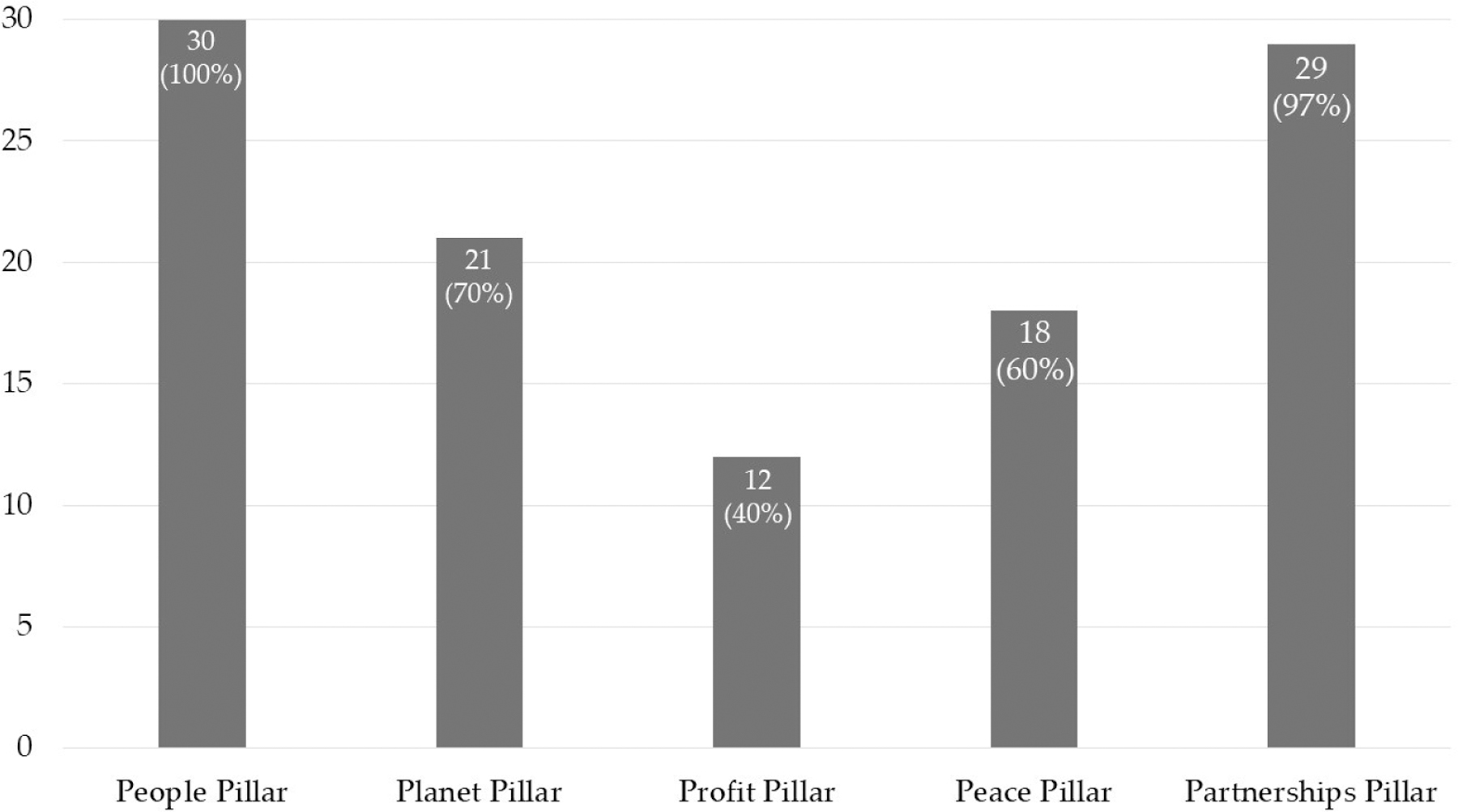
Pillars of Sustainability Identified in Exemplary “Bright Spot”.

**Table 1.** Descriptions of “bright spots” meeting five dimensions of equity, *n* = 30.

Name	Domain	Location	Region	Time Period
Healthy Homes Initiative	Housing	New Zealand	Oceania	2009–ongoing
Canal-side community upgrading at scale	Housing	Bangkok, Thailand	Asia	2003–ongoing
JRMK Cooperative	Housing	Jakarta, Indonesia	Asia	2017–ongoing
Community-led housing and community space development	Housing	Dhaka, Bangladesh	Asia	2020–ongoing
Community-led Water and Sanitation in Kampala’s urban informal settlements	Water and Sanitation/Hygiene (WASH)	Kampala, Uganda	Africa	2014–2020
Enhancing sustainable access to safe clean water and gender-sensitive sanitation services in Epworth	Water and Sanitation/Hygiene (WASH)	Harare, Zimbabwe	Africa	2005–ongoing
Vale Encantado Sustainable Community	Water and Sanitation/Hygiene (WASH)	Rio de Janeiro, Brazil	Latin America	2021–ongoing
The Nuku’alofa Urban Sector Project	Water and Sanitation/Hygiene (WASH)	Nuku’alofa, Tonga	Africa	2011–2020
Healthy Liveable Cities Policy and spatial indicators research program	Governance	Australia and globally	Oceania	2012–ongoing
Observatory of Urban Health of Belo Horizonte (OSUBH)	Governance	Belo Horizonte, Brazil	Latin America	2002–ongoing
Building Healthy Communities (BHC) Initiative	Governance	CA, USA	USA/Canada	2010–2020
Keeping an Eye on Maré|De Olho na Maré	Governance	Rio de Janeiro, Brazil	Latin America	2016–ongoing
District System of Care	Gender Equity	Bogota, Colombia	Latin America	2020–ongoing
Mahila Housing Trust	Gender Equity	Ahmedabad, India	Asia	1994–ongoing
Barka Foundation—Source of Life	Access to Income and/or work	Poland	Europe	1989–ongoing
Long Beach Fresh Crop Swap	Food Systems and/or Agriculture	Long Beach, CA, USA	USA/Canada	2016–ongoing
Herbal and Nutrition Garden in Warren Park	Food Systems and/or Agriculture	Harare, Zimbabwe	Africa	2007–ongoing
Urban Agriculture in Nairobi County	Food Systems and/or Agriculture	Nairobi, Kenya	Africa	2013–ongoing
Placemaking at Mexico	Placemaking	Mexico City, Mexico	Latin America	2019–ongoing
Local Play Everyday	Placemaking	Logan, Australia	Oceania	2020–ongoing
Limeños al Bicentenario: Community recovery of public spaces with an Urban95 approach	Early Childhood	Lima, Peru	Latin America	2019–ongoing
Urban95 Grow with my neighborhood|Crezco con mi barrio	Early Childhood	Bogota, Colombia	Latin America	2017–2019
Kounkuey Design Initiative’s Kibera public space project	Climate Change	Nairobi, Kenya	Africa	2006–ongoing
Cooling Western Sydney: A Quadruple Helix Approach	Climate Change	Western Sydney, Australia	Oceania	2018–ongoing
PowerCorpsPHL	Racial Equity	Philadelphia, PA, USA	USA/Canada	2013–ongoing
Measure A Initiative	Racial Equity	Los Angeles County, CA, USA	USA/Canada	2016–ongoing
Advancing Racial Equity on Planning & policy	Racial Equity	CO, USA	USA/Canada	2022–ongoing
Re-ciclo	Waste Management and Recycling	Fortaleza, Brazil	Latin America	2019–ongoing
Sustainable Waste Management to address flooding in Bwaise III parish slum communities	Waste Management and Recycling	Kampala, Uganda	Africa	2020–ongoing
Participatory Planning and Action by communities and health workers in frontline health services	Healthcare	Lusaka, Zambia	Africa	2006–ongoing

## Data Availability

Research data is available by emailing Dr. Nishita Dsouza, ndsouza@isuh.org.
