# Supplementary material for: Sustainability and Equity in Urban Development (S&EUD): A Content Analysis of “Bright Spots” from the Accelerating City Equity (ACE) Project
Source: Sustainability. Author manuscript; Available in PMC 2023 Dec 26. (PMC10751036; doi:10.3390/su15097318)
Supplement: Table S1 and Table S2 [file NIHMS1901691-supplement-Table_S1_and_Table_S2.pdf]

Supplementary Tables

Table S1. Content analysis of the five dimensions of equity across exemplary bright spots

Bright Spot	Strategies				
	**Distributional**	**Participatory**	**Recognitional**	**Structural**	**Intergenerational**
Healthy Homes Initiative	Housing ventilation and heating within the community was improved based on the findings from the research studies	Social housing sector worked closely with Maori population to improve the housing outcomes. Huge amount of engagement from people living in the houses, building the houses, politicians, etc.	A case control research study was conducted to follow children's and elder's health based on retrofitted housing and found benefits from insulation and heating	Developed housing standards to include better ventilation and heating Program expected to avert estimated NZ$30 million in health care costs over a 3-year period	Research involved individuals of all ages, and housing policy impacted all ages too
Canal-side community upgrading at scale	Homes and canals have been upgraded across these communities. Long-term tenure security was given to these canal-side communities.	Residents across the communities organized themselves into committees and took things into their own hands, mapping their settlements and working with architects to develop designs.	Canal-side communities organized into committees and conducted their own surveys, mapped their settlements, worked with architects to develop designs and plan their upgraded communities	The central government issued a policy to improve the housing along the canal and allocated a substantial budget to do this.	New amenities were introduced by the canals that impacted those of all generations, e.g. kindergarten, welfare centers, etc.

JRMK Cooperative	Communities that were evicted were rebuilt, upgraded, and occupied. Residents were given land ownership.	Members are the main actors of the organization, leading the push for policy change.<br><br>The government, community, and collaborators partnered together to push for policy change in upgrading kampungs	An Urban Poor Network was developed to identify and solve problems within the community by asking members and collectively deciding on a follow-up action to resolve the problem.	Land advocacy resolved cases and land status in the community to ensure the current residents had access to their own land.	
Community-led housing and community space development	Young professionals were trained to continue the pro-people housing and placemaking practice in Dhaka and beyond<br><br>Housing projects were improved, a community garden and a school were built, and the problem of open drainage was tackled.	Poor communities were involved in various actions, ranging from community profiling to carrying out small interventions and building new housing in their diverse contexts. These communities took the lead in determining the direction of development interventions.	The community of Gabtoli City Colony was recognized as being displaced and had many housing issues.		

Community-led Water and Sanitation in Kampala’s urban informal settlements	Impose the hiring of local labor for interventions prioritizing young people, women, people with disabilities to learn skills that contribute to the welfare of their communities.<br><br>Sustainable toilet models designed to suit the local reality of informal settlements.	Community Contracting model as a strategy to build communities agency, ownership, participation and involvement in development efforts.	Local NGO facilitates process to build community consensus, generate data based evidence of the problem (disaggregating data to highlight vulnerabilities) and select implementation areas.	Saving groups contribute with at least 20% of the funds needed for approved interventions, which are paid back to the members afterwards with interest.<br><br>Local initiative led to new policy framework to guide community hiring processes at the national level.	Train local labor, especially young people, women and people with disabilities, to learn skills that contribute to the welfare of the community and generate income.<br><br>Set up Implementation and Management committees with diverse and relevant actors from the local context to lead the initiative.
Enhancing sustainable access to safe clean water and gender-sensitive sanitation services in Epworth	Pilot project program to encourage its wider uptake in the community in a more sustained manner.<br>Participatory: Grassroots-centred programmes focused on WASH, social enterprise development, local economic development, renewable energy and inclusive service delivery for local economic growth.	Grassroots-centred programmes focused on WASH, social enterprise development, local economic development, renewable energy and inclusive service delivery for local economic growth.	The introduction of the technology was informed by an assessment that involved the participation of the local community. The findings were used in designing of the initiative particularly on the need for the active participation and involvement of local communities’ water testing, purification and sanitation innovation.		

Vale Encantado Sustainable Community	Partnership with international organizations and academia to transfer knowledge and technology to practice in building a biodigester system	Community leadership interaction with international organizations and academia to transfer knowledge and technology to practice in building a biodigester system.			
Healthy Liveable Cities policy and spatial indicators research program	Develop a knowledgeable base of cost-effective interventions for healthy, liveable and equitable communities and research translation.	Conduct interviews with local and state government policy makers to identify the challenges faced by the communities they represent. Include stakeholders in advisory board to ensure research and its communication is relevant to practice and has community buy-in.	Identify inequities and make findings accessible for different audiences to empower community advocates to lobby for change. Determine current policy measures, looking at housing affordability, etc.	Engage policymakers with evidence to enact policy reform for healthier, more sustainable cities for future generations. Separate funders supported project components, with work coming together to form a 'super project'.	
Observatory of Urban Health of Belo Horizonte (OSUBH)	Program was implemented to promote physical activity in vulnerable communities.	Training of qualified people from public management, academia and technicians from different areas looking at the intra-urban effects on the lives of citizens.	Storage of primary and secondary intra-city data over time. Data collected throughout the observatory can be used to identify vulnerable communities and issues they face.	Observatory to build and strengthen the research about population's health and conduct studies of urban themes that may guide planning.	

Building Healthy Communities (BHC) Initiative	Community learned how to work with decision-makers, different departments, how to write grants, etc.<br><br>More landscaping, artwork to represent the community, improvements in the community	Community came together and engaged with the decision-makers to learn how to read their own community, identify the problems and possible solutions, and learn how to address different issues related to public health	14 of the most disadvantaged communities in California were identified and demonstrated significant health disparities.<br><br>Provided spaces to ensure the community was comfortable and could participate and vote on what they wanted to see	Adoption of a Health and Wellness Chapter into the City of Coachella's General Plan.	
District System of Care	Locate district blocks ("anchor building") at a distance that ensures services can be accessed by caregivers within a 15-20 minute walk.<br><br>Provide fully equipped vehicles to offer care services in rural and urban areas of difficult access in the city.	Use of "Our Voice citizen science method" to identify the specific needs and curate the services provided.	Recognize the contribution of caregivers and how women's unpaid care work have stopped them from pursuing personal development activities.	Position women, caregivers and care work at the center of planning to organize several of the city's services.	

Mahila Housing Trust	Development of women's grassroots institutions and build their knowledge and capacities to engage with decision-making on issues that concern their communities and cities.<br><br>Provision of technical expertise in construction, technology, and urban governance to help design and deliver pro-poor services and programs.	Act as a bridge between grassroots and mainstream public and private sector institutions and advance action on responsible urban development.	Facilitation of the process of involving women from the communities in identifying the problem to be solved.	Always find women from the community as mobilizers to build community trust and engage them in governance.	
Barka Foundation – Source of Life	Creation of institutions called Work Integration Social Enterprises (WISE) such as the Centers of Social Integration, giving possibilities for job training and education of excluded groups of citizens, social cooperatives, social enterprises, public-social-private partnerships aiming to create more inclusive local markets.			Influence politics to create laws, regulations on social entrepreneurship, establishing regional centres for social enterprises, work integration centres etc.	

Long Beach Fresh Crop Swap	Local leaders created Long Beach Fresh (LBF) to improve the local food system and foster greater social connection among residents through a food swap where residents share their garden-grown fruits and vegetables at a local meet-up.	Connect residents in the food swap through matching and exchanging fruits and vegetables.<br>Support residents to take advantage of these homemade laws	Targeted 3 neighborhoods with the highest levels of chronic disease, including obesity, diabetes and asthma in the California Residents in the neighborhood were surveyed why they weren't eating healthy food and the biggest reason was that they can't afford it.	The California Homemade Food Act was created to encourage local places to sell homemade foods Introduced a law that if a vacant lot will be given over to agricultural/local farming and gardeners, it will be taxed as an agricultural property rate rather than a residential property rate	
Herbal and Nutrition Garden in Warren Park	The initiative has scaled from a community herb garden to 30 gardens with other community members being allocated a portion of land for nutrition gardening.<br>Local collaboration was created with ten schools that benefited by receiving skills, free herbal seeds and the establishment of herbal gardens.	Lack of group cohesion becomes a huge barrier to the continuation of the initiative beyond the donor funding period.<br>Urban initiatives should involve people who have mutual understanding, respect for the founding principles, and agreed constitution to guide the operations of the initiative and clearly outlines the roles, responsibilities, rights and obligations of all involved.		Residents applied for land with the local authority to start an herb and nutrition garden and fishponds avoiding acrimony and enabling community initiatives to be implemented smoothly with the full support of authorities.<br><br>To qualify for funding and technical support, community members joined the initiative so that they could earn a living from selling herbs. This initiative was imposed by the donors and collapsed as soon the funding dried up.	

Urban Agriculture in Nairobi County	Local government in partnership with numerous relevant stakeholders provides food supplements, cash transfers, intersectoral capacity building training sessions, technical assistance and platforms for further engagement, learning, action and advocacy to community focal points/leaders, community-based organizations and farmers groups as well as its own staff from numerous departments.	Local government in partnership with numerous organizations provides and jointly facilitates intersectoral capacity building training sessions, technical assistance and platforms for further engagement, learning, sharing, action and advocacy to community focal points/leaders, community based organizations and farmers groups as well as its own staff.		Research reveals the poor conditions of urban farmers and leads to national urban and peri-urban agriculture and livestock policy with a model for communities, businesses, and government to work together to promote change.	
Placemaking at Mexico	Enhancing early childhood experience and consider their need to turn the public spaces into a place that supports and encourages them in their health, growth, development and education. A playground keeps children active, improving their physical and emotional well-being.	Create fun, safe and participatory public environments designed for children and their caregivers first years of development.	Placemaking methodologies to create public spaces and to strengthen communities around these spaces.	Placemaking methodologies to create public spaces and to strengthen communities around these spaces. 'Backing International Small Restaurants' supports the reactivation and economic recovery of ten independent and culturally significant restaurants in Mexico City	Creation of public spaces that contributes to the development of children.

Local Play Everyday	Children and the communities received free physical activity in the form of sports engagement	local community, government departments, activity providers, etc. Local and Indigenous Elders were involved and became champions for the cause.	Communities chosen based on existing data showing high levels of socio-economic disadvantage. Residents identified key issues facing their community, rather than external individuals identifying issues for them. Children respected and viewed as valued citizens and social actors throughout project to reduce power disparities.		Children experienced increase participation, parents learned about physical literacy development and engagement, elders became champions for the cause
Measure A Initiative	Create safe city parks for all neighborhoods, particularly those in low-income neighborhoods and communities of color. Parks & Rec Department provide classes and events at these parks, beaches, and greenspace, which facilitated social relationships and social cohesion.	Parks department went to every community, hired local community organizations and paid them to host local community meetings to ask what they want to see, building a movement led from the ground up by community and those most impacted by the disparities that exist.	The Parks department targeted communities of low-income and communities of color.	Local funding through the collection of a tax helped build and improve parks, recreation spaces and local water sources within the neighborhood, leading to a collection of ~$96.8 million/year.	Expand funding to reduce gang activity and create safe play areas for future generations.

Limeños al Bicentenario: Community recovery of public spaces with an Urban95 approach	Develop a framework and tested in +20 public spaces with specific goals translated into indicators and expected result to measure the impacts of the project.	Survey with community to engage them into actively participating in neighborhood meetings or neighborhood improvements. Citizen participation, seeking behavioral change, generating and using data for decision-making (and storytelling), maintaining agile/immediate (tactical) transformation	Selection criteria of areas of interventions based on neighborhoods of high social vulnerability.	Review services provided by the local government (in isolation) and use existing financial resources in the areas of maintenance, social promotion and others to design and test a framework for the immediate and participatory recovery of public spaces for local governments.	
Urban95 Grow with my neighborhood | Crezco con mi barrio	Delivery of child friendly public spaces using concepts and tools provided by international organizations.	Use of strategies to engage children and caregivers in decision-making.	Use of existing data (10 indicators) to create heatmaps and visualise where young children face the greatest challenges.	Identify current and planned official interventions to work alongside such interventions to offer greater potential opportunities to create a feeling of change in which the Urban95 project could leverage.<br><br>Pilot Project influenced the development of new city wide policies.	Creation of public spaces that contributes to the development of children.<br><br>Local government joined a global initiative led by Bernard van Leer Foundation to make cities child friendly (Urban 95).

Kounkuey Design Initiative's Kibera public space project	Provide multipurpose public spaces that Improve access to essential services and enhanced flood protection infrastructure and resources for residents.	Local organization organizes sessions, meetings, trainings and interactions for Community members, youth, leaders and organisations to lead and facilitate the planning and changes, designing and implementing carefully researched and tested approaches to improve livelihoods and help mitigate flood risk.	Create a platform with information about weather/climate available to residents of informal settlements to help them better prepare for rainy episodes and seasons.	Highlight the necessity of state intervention to invest in infrastructure (waste collection, urban agriculture, etc) to subsidise services and land use for low income communities, or to set legal standards for private developers that protect the interests of low income communities.	Create training opportunities and jobs for youth and created support systems that allow women with children to balance work and childcare. Promote collaboration between communities and various state and non-state actors to get contributions to mitigate the numerous environmental, social and economic challenges.
Cooling Western Sydney: A Quadruple Helix Approach		Multi-sectoral collaboration and investment from government and public sector, academia, community, and industry partners.	Existing data shows vulnerable communities in Western Sydney is hot and is set to get hotter due to a combination of climate change, local geography, and rapid urbanization. Sweltering cities community survey was launched, with respondents aged from under 18 - over 80 describing what they experience with heat in their homes.	Initiative led to four different reports that laid out recommendations for introducing climate sensitive design and planning strategies and improved heatwave management.	

PowerCorps PHL	Out-of-school or out-of-work 18-30 year olds are engaged in an immersive, paid 4-24 month program that results in connection to living wage jobs in energy, green infrastructure, and community-based careers.		These individuals targeted for the program are identified as the most impacted by the city's gun violence and lack of opportunities		Youth can begin in this program to gain work experience and continue to work in this field as they get older and enter adulthood
Keeping an Eye on Maré | De Olho na Maré			Inclusion of favela residents and organizations in data collection that contribute by reporting and validating evidence on the violence that has occurred. Stimulates reflections and generates data on the public security model in favelas and urban peripheries, the profile of victims of violent lethality (young people, black people, favela residents) and the city model (segregated, unequal, polarized, violent).	Use of data to highlight the impacts of urban violence on underserved communities and guide the development of plans and policies.<br><br>Promotion of studies and reflections on the predominantly adopted public security model.	

Advancing Racial Equity on Planning & Policy	Apply, adapt, and grow the Racial Equity Toolkit use and integrate the Toolkit into project development processes.	Compensate community members during engagement and survey those who rely on alternative mobility.<br><br>Establish ongoing transportation equity advisory committee.	Document and share histories of Denver's BIPOC communities and transit deserts.<br><br>Grow and refine racial equity data sources.	Plan will guide long-term infrastructure investments to evaluate and assess throughout the lifecycles of this project.	
The Nuku'alofa Urban Sector Project	Upgrade living standards of the residents with better infrastructure and services.	Community workshops, meetings and engagements were held with affected parties, wider city citizens.<br><br>Involved more women in decision-making processes and leadership roles.		Improved water monitoring to ensure clean water access to residents, improved waste collection and stagnant water to reduce vector-borne diseases, improved safety for residents by using sidewalks and crossroads to reduce traffic injuries and introduced street lighting, gather strong enough evidence (data) to influence policy change.	

Re-ciclo	Pilot Project to test new type of benefit (electric cargo tricycle), being adjusted according to target audience feedback.<br><br>Data collection to demonstrate impacts of the Pilot Project.	Provision of trainings seeking the autonomy of the waste collectors and sustainability of the project.	Group-specific data collection.<br><br>Focus group and technical visits to understand the demands of the group.	Specific regulation to allow the local government to provide fair benefits.<br><br>Proposed solution linked to a topic with visibility in the city: Fortaleza was very prominent in Sustainable Mobility. The project links mobility and waste management, achieving greater validation from decision makers.	
Sustainable Waste Management to address flooding in Bwaise III parish slum communities	Address a community issue (floods) with a solution that generates jobs and income for the most vulnerable.	Continued stakeholder engagement during implementation as an approach to continually generate people's ideas and insights, revise plans and allows for two-way accountability between TAU and the communities (enhances buy-in by the different actors within the community)	During the inception mappings and stakeholder engagements, the initiative gathered evidence on the “who” was most affected, “where” they lived, and “why” they were affected. This informed the plans to ensure that those suffering the most from flooding and its effects were prioritized.	Coinciding the initiative's defense with the election period to keep leaders, including aspirants, engaged and interested in listening to communities' demands.	

Participatory Planning and Action by communities and health workers in frontline health services	Participatory, reflection and action (PRA) tools raise awareness of the constraints faced by health facilities in reallocating the meager resources that are allocated from the district and central level, and help HCCs to better target these resources towards the specific needs identified by community members.		Photovoice is a Participatory Action Research (PAR) tool that combines photography with community action by putting the camera into the hands of community participants so they can represent their community's point of view through the visual images.<br>Participation, reflection and action (PRA) methods are used to build a shared identification of problems and remediation.	Participatory approaches targeted at influencing decision makers to integrate the Social Determinants of Health in their planning and budgets by providing them with photographic evidence of the reason for change being advocated.<br><br>Documenting the work helped to build trust in and respect for the work by different institutions. Reporting also helped with transparency on the processes.	

Table S2. Qualitative content analysis of the five pillars of sustainability across exemplary bright spots

Bright Spot	People Pillar	Planet Pillar	Profit Pillar	Peace Pillar	Partnerships Pillar
Healthy Homes Initiative	Social housing sector worked closely with Maori population to improve housing outcomes, and there was a huge engagement from people living in these houses to work with policy makers.	Building standards for new buildings were improved based on this project, standards for social housing were improved, homes were future-proofed for climate change		While this group constitutes a small population size, Maori are disportionately represented in rural areas. Increased investment in Maori housing is occurring to remedy this problem.	There are examples of formalized governance structures established between the public housing provider, local government, and iwi/Maori.
Canal-side community upgrading at scale	Residents secured land tenure (collective 30-year renewable land leases) and at less risk of eviction. In addition, their housing was upgraded and the canals beautified. The process was community-driven, and resulted in significant improvements to individual and community health.	Improving the pollution/flooding of canals by rebuilding informal settlements in these areas. Creation of gardens and green communal spaces in canals. Canal revitalization has led to improvements in ecology, such as cleaner water and catfish returning to canals.	Pilot projects --> 12 communities, scaled up to 50 communities and 10 canals, given a special budget because "clean canals" were made a political priority. MOU between CODI and Treasury Department allowed for long-term land leases to community groups. New opportunities for economic development, such as community tourism and shops/home-based businesses.	Focus population is squatter settlements / urban poor that live in that area	Good relationships between the government, local governments, and community members, as well as acceptance from the government to be a part of the city's growth (Collaboration between people – public – private – academic)

JRMK Cooperative	Initial formation of savings groups to socially organize, establishing a new power structure by women. Followed by cooperative structure to increase economic empowerment, provide better services to members, and serve as a collective legal subject. Internal mechanisms allowed for participation in decision-making and women empowerment. coops have established collective land rights and future tenure security. Social ties and networks strengthened because of coops.	Kampungs that were physically upgraded now have better and safer access to clean water, sanitation systems, and waste management. Simultaneously, the availability of these services implies less pollution in the environment.	Initial formation of savings groups, to collect funds but also socially organize. Cooperative structure to obtain legal titles for land, access public budgets, and improve bargaining position with governments. Coop manages funds through business units, which help secure needed resources (e.g., rice, liquid fertilizer, etc.), with the business revenue distributed among members and funding organizational activities. Coops have established collective land rights and future tenure security.	JRMK organize kampungs; therefore, the urban poor can exercise the right to their city by fighting against the threat of forced eviction and have legal certainty to live. Organizing also promotes cultural change, one of which is gender equality. The organizing by JRMK Jakarta pays attention to women's groups, where women are encouraged to be vocal and participate in the decision-making process.	Through networking, JRMK builds the bridge of cooperation and has shown the growth of partisanship between classes. Through collaboration, middle-class professionals work together with kampung residents to solve city problems. These middle-class professionals include lawyers, architects, journalists, academics, students, artists, NGO activists, and environmental activists.

Community-led housing and community space development	Adults in the community worked on housing, children banded together to launch their own initiatives, and women were responsible for forming the savings group. A local market was run by Colony residents in the neighborhood. The savings group became the foundation of how processes were built, including community mapping, design workshops, housing construction	The Colony was built near the Buriganga River and Gabtoli bus terminal, on a garbage-filled site with no roads, water, or other infrastructure. Over the years, NGOs and local governments helped built roads, water lines, and toilets, but these were far from adequate for the settlement's population.	A citywide housing fund was established by the community dwellers to be used as low-interest loans to support additional housing projects.	The project has had a significant impact on women, in particular, who have led the process from start to finish. The formation of savings groups has strengthened their unity and increased their awareness of their collective power. Many women were even involved in construction. Their accomplishments and stories have inspired other women in the community and serve as a model for the country.	In addition to these key actors and partners, the Housing and Building Research Institute of Bangladesh has contributed technical resources on material and construction technology. Local community and political leaders, as well as ward administrators, intervened to resolve conflicts and find mutually acceptable solutions, while local development authorities assisted in working around bureaucratic and political processes. Finally, private design and development firms in Bangladesh, such as Marina Tabassum Architects and Suvastu Developers, have volunteered their structural building knowledge to the communities.

Community-led Water and Sanitation in Kampala's urban informal settlements (5) – Kampala, Uganda	build capacity of the urban poor to improve their livelihoods and increase their access to basic social services and resources (land housing). The overall aim of this initiative was to build resilience and improve the water and sanitation of the urban poor communities and slum dwellers.<br><br>Improved sanitation resulted in less diarrheal disease		Over 930 local youth (245 women, 695 men) have acquired skills through an apprenticeship with contractors during the construction of the toilets. These form a pool of human resources for future local contracting approaches.	The user committee (PMC) establishes guidelines to ensure that all individuals are served equitably. Examples include:<br>• Children 0-13 years or people with disabilities<br>not were not required to pay.<br>• Jobless youth, women and any adults whose were unable to pay either daily or monthly pay in kind (for example through contribution to cleaning, emptying of sludge,<br>maintenance etc.	Federal government initiative, local government actors, formation of committees
Enhancing sustainable access to safe clean water and gender-sensitive sanitation services in Epworth	Residents participated in learning platforms to improve hygiene practices and engaged and worked with local authorities to identify solutions to some of the challenges in WASH.	Thirty pit latrines were installed in Epworth, Harare, Zimbabwe using Easy-flush toilet flushing systems that uses only 2 liters of water compared to the conventional flushing systems with 9 liters. Reduced water usage allows elimination of strain to the municipal treatment plants.			Civic Forum on Human Development (CFHD) in partnership with UN-Habitat, Santinton Contractors and community based organizations working in Epworth worked with residents.

Vale Encantado Sustainable Community	Social equity component; fostered wellbeing	The impacts generated in the community:<br><br>• Low cost water heater<br>• Ecological walk with public and private schools<br>• Recreation area<br>• Garbage collectors (biodigester)	Focus on financial stability for residents by creation of community-owned waste management system		The residents of Vale Encantado – collaborating with engineers from the local University, international partners, and experts in biodigester construction – built their own biodigester and waste collection system from local materials.
Healthy Liveable Cities policy and spatial indicators research program	The goal was to tie research to policy through deep engagement with policy makers. Key findings from the research was summarized in policy briefs, contributing to recommendations from the research embedded in other policies. There has also been successful capacity building of local collaborators working with policy makers to amplify findings through local launches and town hall events.	Strategies and recommendations were identified from the research to promote more walkable neighborhoods, resulting in less car pollution			International collaboration among public sector and academic researchers

Observatory of Urban Health of Belo Horizonte (OSUBH)	Increase in physical activity and leisure for residents where programs have been implemented. Monitoring the COVID-19 pandemic: enormous social inequality was revealed in the city, showing a greater occurrence of hospitalization of children aged 0 to 11 years living in vulnerable areas. Homicide rates in informal areas (favelas) that have undergone urban transformations over time have decreased.				The Observatory was founded in 2002 through a collaboration between the Federal University of Minas Gerais (UFMG) and the local government of Belo Horizonte as part of a wide agenda to strengthen the local and national health systems. The observatory works with the training of qualified people from public management, academia and technicians from different areas (public health and other health specialties, geographers, architects, engineers, economists, statisticians).

Building Healthy Communities (BHC) Initiative	The community came together and engaged decision-makers to learn how to read their own community, identify problems and possible solutions, and learn how to address different issues related to public health. They learned how to work with different departments and how to write grants.	The project provided more landscaping, bike lanes, and artwork to represent and improve the community.			Collaboration of 12 policy departments in Eastern Coachella Valley
District System of Care	provided care opportunities to people who need care (e.g., children, elders, those living with disabilities) and provided relief to caregivers			provided equitable opportunities to unpaid caregivers, mostly women, to try to achieve gender equity by relieving them of their care duties for some time	intersectoral strategy of different local governmental entities (secretary of women; social integration secretary; secretary of culture, recreation, and sports; secretary of health; secretary of economic development; secretary of education; secretary of environment)

Mahila Housing Trust - India	MHT connects vulnerable women to the information and tools they need to address their most pressing challenges. Women from low-income communities are empowered to exercise their rights and collectively demand improved living and working environments. MHT builds social capital and community capacities to solve problems in the urban environment.	MHT has helped over 53,000 households with accessing potable water, 59,000 households accessing toilets, 13,000 households benefitting from water management, 80,000 households accessing grid electricity, 44,000 households investing in energy savings products. They continue to promote climate resilience and develop a new kind of roof construction that could reduce indoor temperatures up to 6C. The women have also installed solar panels, reducing average monthly bills by up to 50%.	Women were trained as mobilizers to build community trust and engage in governance. They were also trained on how to initiate conversations with government contracts to receive more money for public resources. About $17 million government contracts were received and $1.4 million saved by credit co-op members in the community.	Strong gender equity focus, with empowering women.	Asian Coalition for Housing Rights (ACHR) collaborates with e collaboration between Mahila Milan, the National Slum Dwellers' Federation (NSDF), and the Society for the Promotion of Area Resource Centers (SPARC)

Barka Foundation – Source of Life	solidarity and togetherness can be the basis for integrating marginalized citizens and they are social entrepreneurship pioneers who do their work with devotion and belief in a higher quality life, basing their achievements on the self-empowerment and leadership principles, making changes within, and inspiring community, Ministries, companies to use social entrepreneurship concept to ensure a higher quality of life for all citizens		Main goal is socio-economic integration / empowerment of marginalized groups and with this goal, come other equally relevant goals: Development of social entrepreneurship for community economic empowerment Changing / influencing policies for social integration, designing laws and strategies on social entrepreneurship (currently in Macedonia)	Barka promotes support to and empowerment of the following marginalized groups, regardless of their age, sex, religion – the homeless, former addicts, persons with disabilities, immigrants and refugees, Roma communities etc. by providing re-integration programs through social entrepreneurship development	Collaboration between Various Ministries in Poland and in other countries where Barka operates in the EU / SEE region (Macedonia), Canada and Africa; Local municipalities in Poland and other countries / with local structures: social enterprises, civil society, companies, social centres, education entities etc.; and Marginalized group representatives / Barka leaders to ensure ownership and self-empowerment
Long Beach Fresh Crop Swap & Food Policy Council	Local leaders created Long Beach Fresh (LBF) to improve the local food system and foster greater social connection among residents, improve access to healthy food, support businesses, improve procurement by schools, and reduce food waste.	A policy was established to introduce community gardens in large public parks and incentives were created to use vacant lots for urban agriculture.	Urban agriculture vacant lots created economic opportunities		LBF has collaborated with City Council. LBF and 22 other food policy councils, are members of a statewide organization called the California Food Policy Council. A national model for community driven healthy food access and social connection.

Herbal and Nutrition Garden in Warren Park 2	Community gardens can mitigate some of the problems that plague urban areas such as urban poverty, malnutrition and unemployment. They can be a beneficial addition to many communities by increasing the availability of nutritious foods, strengthening community ties, reducing environmental hazards, reducing food miles and creating a more sustainable system (Toriro et al.,2005) On the other hand, community gardening offers a unique avenue to well-being through engagement in activities and responsibilities that encourage social cohesion, skills acquisition, and personal growth.			Residents in Warren Park two are highly poverty stricken as they face challenges such as high food prices, accommodation, user fees for water and electricity, and associated debt. The situation is compounded by high unemployment and low economic activity. Poverty is widespread in Warren Park urban and the area is fast becoming increasingly worse off	Collaboration was initiated with institutions in USA, Germany, Sweden and Africa University with interest in herbs, and collaborators used to come from these countries and institutions to support the initiative. Local collaboration was also created with ten schools that benefited by receiving skills, free herbal seeds and the establishment of herbal gardens.

Urban Agriculture in Nairobi County	The Nairobi and Environs Food Security, Agriculture and Livestock Forum (NEFSALF) provided communities, networks of farmers, and the public and private sectors a platform to engage, deliberate, and take action on issues concerning agriculture, livestock and food security.	Farming practices were taken up to address water and land access, soil quality, and highlight and mitigate climate change while continuing to feed the population. They focused on improving land management and design of settlement areas; land use systems that integrate trees, shrubs, or crops; farming methods that involved both raising of livestock and cultivation of crops, with the dung from livestock often being used as fertilizer, and urban farming methods and aquaponic food systems.	Project was funded by the local government and partnerships. The project aimed to promote improved and most sustainable urban agricultural practices in informal settlements, including cash transfers, supply of food supplements, development of adaptable business models, etc.		The county council has partnered with several organizations including FAO, the World Bank, Mazingira Institute, community leaders and community based organizations from informal settlements and other stakeholders to facilitate this by promoting urban agriculture especially in informal settlements; by highlighting and addressing the factors that affect urban agriculture including soil quality, water and land access and climate change; and also by streamlining their own internal operations to improve service delivery.

Placemaking at Mexico	Aims to enhance early childhood experience and consider their need to turn the public spaces into a place that supports and encourages them in their health, growth, development, and education. A playground keeps children and their caregivers active, improving their physical and emotional well-being	Placemaking is often done with natural or recycled materials.			FEMSA Foundation and Placemaking Mexico Foundation, IDA Foundation, AMEX, and frequent collaboration with government and local institutions

Local Play Every Day	The local community engaged, coordinated, and collaborated with local government departments, and existing activity providers to facilitate play-based supportive activities for local children and families. Children were viewed as a valued citizen and social actor. Ongoing local play friend networks were created that enhanced connectivity, cohesiveness, friendliness and perceptions of safety within local neighborhoods. Local and indigenous wisdom was honored and local Elders were actively involved, becoming champions of the cause.			In 2019 Logan City Council had seven suburbs with high levels of socio-economic disadvantage, ranging from 75-100% of the highest levels of disadvantage in the Index of Relative Socio-Economic Disadvantage. This comprised 25% of the local population in Logan (James, 2019).<br><br>The residents of the Logan area are culturally diverse, with 34.2 % of the population being born outside of Australia (Australian Bureau of Statistics, 2017).	Logan Together partnered with Queensland Governments Department of Sport and Recreation, later transferred to Australian Institute of Play

Measure A initiative	All will benefit, but focus will be on communities with most park need (low-income and communities of color). Mental health benefits of greater access to public green and blue spaces. Community-driven effort (conducted parks accessments, advocated for what they wanted to see in the area). Oversight structure in policy to ensure accountability and community engagement.	Support for local parks, beaches, open space, and water resources (conserve and protect local water sources and undeveloped natural areas).		Strong racial equity focus of the measure, primarily focuses on those communities with the most park need (e.g., low-income communities and communities of color)	Required county to interact with all cities in the council, local govt is involved. here are a number of key adovacy groups in Los Angeles who were part of the campaign to get this passed. Also, Supervisor Hilda Solis was the Measure's champion and her staff are available. Finally, the department of parks and recreation would be good to speak with on the Measure.
Limeños al Bicentenario: Community recovery of public spaces with an Urban95 approach	Neighbors had articulated an agenda and/or had an active initiative with actions to improve their public spaces. The community was engaged to actively participate in neighborhood meetings or neighborhood improvements	Infrastructure improvements included increased safe pedestrian area from motorized vehicles, increase of natural elements, urban green and environmentally responsible practices, increase in elements for rest and play,		Interventions impacted neighborhoods of high social vulnerability with risks for babies, children and their caregivers.	The local government used existing financial resources in the areas of maintenance, social promotion, and others, and identified the actors to be engaged that were already carrying out the proposed activities, but in isolation.

Urban95 Grow with my neighborhood | Crezco con mi barrio	Guaranteeing healthy, safe and friendly urban environments to children and families, opportunities for their personal development and a better quality of life	Environmental upgrading with green infrastructure, a land ordinance plan; public space policy; and parks, promenades and plazas handbook		Areas targeted based on indicators such as: Children living in poverty and extreme poverty; the rate of children under-5 Y.O. served by welfare programs; the rate of 5 Y.O. enrolled in kindergartens; chronic mal nutrition, mortality and vaccination; adolescent birth rate; domestic violence; and population density, crime in public space and public space m2 per inhabitant.	A pilot project was developed based on an interagency collaborati on approach + participatory planning
Kounkuey Design Initiative's Kibera public space project	Kounkuey Design Initiative is a community development organisation founded by local students. They worked as consultants with residents and community organisations to design interventions to plan and build on underutilised sites.	Eleven new climate-resilient public spaces and their infrastructures were established, a solar powered water heating system for the sanitation business, a rainwater harvesting and storm water management system, and installation of Wi-Fi facilities inside the community hall were also introduced.			KDI partnership with community organizations, residents, agencies, CSOs, NGOs, governments and universities to advocate for improved policy and practice for residents of Kibera.

Cooling Western Sydney: A Quadruple Helix Approach	Sweltering Cities to survey communities impacted by extreme heat to secure cooler, more equitable and sustainable cities, with planning and policy that puts people at the center.	Addressing urban heat island in Western Sydney by using greenspace and water			Co-designed by over 55 stakeholders, with 4 major players (1) government and public sector, 2) academia & universities, 3) community/civil society, and 4) and industry/business partners. Multi sectoral collaboration and investment.
PowerCorps PHL	PowerCorpsPHL engages out-of-school or out-of-work 18- to 30-year-olds. Additionally, they recruit young people most impacted by the city's gun violence and lack of opportunities.	The jobs within PowerCorpsPHL are focused in energy, green infrastructure, and community-based careers. To date, they have provided over 800,000 service hours to the city's park system and green infrastructure.	Over 90% of graduates transition into employment with starting wages ranging from $13-$20/hr. This partnership has yielded 70% of PWD's apprenticeship spots being sourced from PowerCorpsPHL talent for high-need positions identified by the water utility. In their eight years of operations, PowerCorpsPHL has engaged over 800 young people and provided over 800,000 service hours to the city's park system and green infrastructure.	Strong racial equity focus	Collaboration between City of Philadelphia and AmeriCorps

Keeping an Eye on Maré ǀ De Olho na Maré	Creating a model of public safety to avoid militarized childhoods and lessen the intergenerational impacts of violence.			Focused on a favela population, increasing equity by lessening trauma among urban underserved	a network of collaborators, with 197 residents and 21 organizations active in the favelas of Maré
Advancing Racial Equity on Planning & Policy	The toolkit is designed to help staff, in collaboration with communities of color, deliver projects and programs equitable across transportation. The project team used a variety of methods to assess racial equity understanding and applied practices within the DOTI, including focus groups with DOTI staff, peer city and expert interviews, a staff survey, and research on emerging equity practices.			Hire BIPOC staff and contractors. Continue and maintain staff racial equity training. Compensate community members during engagement.	

The Nuku'alofa Urban Sector Project	Upgrading living standards of Nuku'alofa residents with urban necessities (water supply, sanitation, waste management), resulting in improved health outcomes.	Zones are prone to flooding, stagnant water, and poor sanitation; project focused on future-proofing urban areas to mitigate impacts of climate change and other natural disasters.		Significant focus on gender equality in terms of participation, capacity building, and decision making	political leadership with third parties (national and multilateral funding) (involved - Prime Minister of Tonga (Governance leadership); Government Ministers (project level with regard to their responsibilities; Asian Development Bank (project funder, project leadership); Australian Government (co-funder); Third party experts (Tonga and oversees) for specific project areas; Local communities (business, social services, residents); women's groups

Re-ciclo	Focus groups and technical visits occurred to understand the demands of the waste collectors. Local government worked with waste collectors, and partnerships with startups, social impact companies and private sponsorship occurred to scale the project.	Twenty electric cargo tricycles were given to 40 waste collectors who went around to the community to collect and recycle materials. This led to greater interest of the population in separating recyclable materials and elaboration of new local public policies including waste collectors.	Waste collectors' income increased by 40% during the pilot project.		Creation of specific regulation, in accordance to the National Policy of Waste Management, to allow local government to work with waste collectors, providing the benefit (electric cargo trycicle and other services) Fundraising and technical partnership (with international organizations - CAF and GIZ) through submission of a proposal in an international call (TUMI) for a pilot project. Partnership with startups, social impact companies and private sponsorship to scale the project

Sustainable Waste Management to address flooding in Bwaise III parish slum communities	Improves health of urban slum dwellers by reducing their exposure to floodwaters with waterborne diseases	Reduce flooding caused by poor disposal of waste that gets mixed in with flood water run-off, by turning waste into marketable products	Solid waste briquettes for a living and reducing domestic energy poverty by using briquettes for cooking	Targeted the most affected populations (e.g., women and children)	Partnership between NGO and local leaders, engaged 40+ key stakeholders from district public health offices and local leaders
Participatory Planning and Action by communities and health workers in frontline health services	Through meetings and training sessions, community exchanges and interactions take place with frontline health workers about health needs and gaps or service barriers. This information is used to arrive at shared action plans with prioritized issues. Community members were trained by the Lusaka District Health Office (LDHO) in PRA methodology and processes to demystify and progressively increase community dialogue/health literacy.				Community members worked in their zones to implement the strategies identified in collaboration with civic leaders and with the Local Authority.
